# *In-Utero* Low-Dose Irradiation Leads to Persistent Alterations in the Mouse Heart Proteome

**DOI:** 10.1371/journal.pone.0156952

**Published:** 2016-06-08

**Authors:** Mayur V. Bakshi, Omid Azimzadeh, Juliane Merl-Pham, Tine Verreet, Stefanie M. Hauck, Mohammed A. Benotmane, Michael J. Atkinson, Soile Tapio

**Affiliations:** 1 Helmholtz Zentrum München, German Research Center for Environmental Health GmbH, Institute of Radiation Biology, D-85764 Neuherberg, Germany; 2 Helmholtz Zentrum München, German Research Center for Environmental Health GmbH, Research Unit Protein Science, D-80939 Munich, Germany; 3 Chair of Radiation Biology, Technical University of Munich, D-80333 Munich, Germany; 4 Radiobiology Unit, Belgian Nuclear Research Centre, SCK-CEN, B-2400 Mol, Belgium; 5 Laboratory of Neural Circuit Development and Regeneration, Animal Physiology and Neurobiology Section, Department of Biology, KU Leuven, B-3000 Leuven, Belgium; University of Arkansas for Medical Sciences; College of Pharmacy, UNITED STATES

## Abstract

Prenatal exposure to stress such as increased level of reactive oxygen species or antiviral therapy are known factors leading to adult heart defects. The risks following a radiation exposure during fetal period are unknown, as are the mechanisms of any potential cardiac damage. The aim of this study was to gather evidence for possible damage by investigating long-term changes in the mouse heart proteome after prenatal exposure to low and moderate radiation doses. Pregnant C57Bl/6J mice received on embryonic day 11 (E11) a single total body dose of ionizing radiation that ranged from 0.02 Gy to 1.0 Gy. The offspring were sacrificed at the age of 6 months or 2 years. Quantitative proteomic analysis of heart tissue was performed using Isotope Coded Protein Label technology and tandem mass spectrometry. The proteomics data were analyzed by bioinformatics and key changes were validated by immunoblotting. Persistent changes were observed in the expression of proteins representing mitochondrial respiratory complexes, redox and heat shock response, and the cytoskeleton, even at the low dose of 0.1 Gy. The level of total and active form of the kinase MAP4K4 that is essential for the embryonic development of mouse heart was persistently decreased at the radiation dose of 1.0 Gy. This study provides the first insight into the molecular mechanisms of cardiac impairment induced by ionizing radiation exposure during the prenatal period.

## Introduction

Ionizing radiation is now recognized as a risk factor for cardiovascular disease [1]. It is suggested that children may be more susceptible to future radiation-induced heart impairment than adults [2]. Given the well-documented association between prenatal radiation exposure and both childhood cancer [3] and developmental impairments [4,5], the incidence of hypertension, hypercholesterolemia and cardiovascular disease was investigated in atomic bomb survivors exposed *in utero* [6]. Although no significant excess in the risk for cardiovascular disease was found, there was a suggestion of an increased risk when fatal and nonfatal cardiovascular disease cases were combined. This study has statistical limitations due to the low number (506) of *in-utero* exposed subjects studied and their relatively young age (under 60 years) at the time of examination [6].

The embryonic development of the murine fetal heart is a very dynamic process as the typical gestation period only lasts three weeks. A. particular window of susceptibility extends from embryonic day E9 to E12. When corrected for size and embryonic time scale, the anatomy and growth of mouse and human hearts are quite similar [7], suggesting that mouse models can be successfully used to study the heart development.

The activation of the MAP kinase cascade plays an important role in the heart chamber formation of the fetus [8]. Data from human studies suggest that environmental stress factors such as hypoxia, increased level of reactive oxygen species or antiviral therapy adversely influence this process [9,10]. It is reasonable to suggest that ionizing radiation may have similar adverse effects on the fetal heart development. However, no experimental data concerning radiation-induced cardiac defects after prenatal exposure are available at the moment.

The aim of this study was to evaluate the long-term consequences of low and moderate *in-utero* (E11) radiation doses using the C57Bl/6J mouse model. The lowest radiation dose used here is comparable to that received in coronary computed tomography angiogram (20 mGy). The higher doses of 0.1 and 1.0 Gy have been measured in accidental and occupational situations, even in females [6,11]. Both male and female offspring were included in the study. A global quantitative proteomics analysis with a liquid chromatography-tandem mass spectrometry (LC–MS/MS) identification of dysregulated proteins was performed. Proteins altered in expression could be clustered to several categories including mitochondrial, acute phase and structural proteins. Significant proteome alterations were detectable two years post-irradiation even after exposure to a dose of 0.1 Gy.

## Materials and Methods

### *In-utero* irradiation

All animal experiments were performed in accordance with the European Communities Council Directive of November 24, 1986 (86/609/EEC) and approved by the local ethical board Studiecentrum voor Kernenergie-Centre d'Étude de l'énergie Nucléaire/Vlaamse Instelling voor Technologisch Onderzoek (SCK-CEN/VITO) (ref. 02–012). C57Bl/6J were purchased from Janvier (Bio-services, Uden, The Netherlands) and housed under standard laboratory conditions (12 h light/dark cycle). Mice were mated during a 2-hourtime period in the morning, at the start of the light phase (7.30 h until 9.30 h), in order to ensure synchronous timing of embryonic development. Subsequently, pregnant females were whole body irradiated at E11 (0.02/0.05, 0.1 and 1.0 Gy) at a dose rate of 0.35 Gy/min using a Pantak RX tube operating at 250 kV, 15 mA (1 mm Cu-filtered X-rays). The calibration of the X-ray tube was performed using an ionization chamber. Control pregnant females were sham-irradiated. The overall health of the mice was monitored weekly. No adverse health consequences were observed in the irradiated mice. All mice were sacrificed via cervical dislocation, the male offspring after 6 months and the female offspring 2 years after birth. Hearts were excised and maintained at -80°C until further analysis. The time points referred to in this paper (6 m, 2 y) are calculated from the birth, not from the irradiation.

### Isolation of total heart protein

The frozen hearts were pulverized in liquid nitrogen using mortar and pestle. The powdered tissue was immediately suspended in 6 M guanidine hydrochloride (SERVA) with phosphatase and protease inhibitor cocktails (Roche). The samples were centrifuged at 13,000 g and the supernatants were collected. Protein concentration of each supernatant was measured with Bradford assay [12].

### ICPL labeling

Fifty (50) μg of protein from each supernatant was labeled with Isotope Coded Protein Label (ICPL) technology. Triplex set of labels (light, medium and heavy) were used as follows: control was labeled with light labels while irradiated heart proteomes (0.02/0.05 Gy, 0.1 Gy) were labeled with medium and heavy isotopes as described previously [13]. Duplex set of labels was used for the dose of 1.0 Gy: control was labeled with a light label and the irradiated sample with a heavy label. Three biological replicates were used for each dose and for the respective control groups. The labeled proteins were mixed, precipitated and dissolved in Laemmeli sample buffer [14]. The proteins were separated by SDS-PAGE gel electrophoresis and stained using Coomassie Blue.

### Mass spectrometric analysis

Mass spectrometric analysis was done as described previously [15]. Shortly, the Coomassie Blue stained protein lanes were cut into 5 slices and individually in-gel digested with trypsin (Sigma Aldrich). The digested peptides were fractionated on nano-HPLC and subsequently analyzed with an Orbitrap-XL mass spectrometer (Thermo Scientific) as described previously [16].

The MS/MS spectra were searched against the Ensembl mouse database (Version: 2.4, 56 416 sequences) using the Mascot search engine (version 2.3.02; Matrix Science) with the following parameters: a precursor mass error tolerance of 10 ppm and a fragment tolerance of 0.6 D. One missed cleavage was allowed. Carbamidomethylation was set as the fixed modification. Oxidized methionine and ICPL-0, ICPL-4 and ICPL-6 for lysine residues and N-termini of peptides were set as the variable modifications.

Data processing for the identification and quantitation of ICPL-triplex labeled proteins was performed using Proteome Discoverer version 1.3.0.339 (Thermo Scientific). The MASCOT Percolator node-based algorithm was used to discriminate correct from incorrect peptide spectrum matches. The q value of the percolator algorithm was set to 0.01 representing strict peptide ranking. Thus, only the best ranked peptides were used. Further, these peptides were filtered against a Decoy database resulting in a false discovery rate (FDR) of each LC-MS-run; the significance threshold of the FDR was set to 0.01 to ensure that only highly confident peptides were used for protein quantification [17]. Proteins identified by at least two unique peptides in two out of three biological replicates, and having an H/L variability of less than 30% were considered for further evaluation. Proteins identified by a single peptide were manually scrutinized and regarded as unequivocally identified if they fulfilled the following four criteria: (a) they had fragmentation spectra with a long, nearly complete y- and/or b-series; (b) all lysines were modified; (c) the numbers of lysines predicted from the mass difference of the labeled pair matched the number of lysines in the given sequence from the search query and (d) at least one mass of a modified lysine was included in the detected partial fragment series [18]. Proteins with ratios of H/L label greater than 1.30-fold or less than 0.769-fold were defined as being significantly differentially expressed.

### Access to raw data files from LC-MS/MS runs

The raw data files from the mass spectrometry analysis have been deposited under the following link-http://storedb.org/store_v3/study.jsp?studyId=1019

### Bioinformatics analysis

Protein-protein interactions and signaling networks were searched using INGENUITY Pathway Analysis (IPA) (http://www.INGENUITY.com) [19] and STRING protein database (http://string-db.org) [20]. The Ensembl protein accession numbers, including the relative expression values of all significantly deregulated proteins, were uploaded to IPA and STRING to elucidate possible interactions.

### Immunoblotting analysis

Immunoblotting of protein lysates from control and irradiated tissues was performed as described [21]. In short, proteins were separated using 1D gel electrophoresis and transferred to nitrocellulose membranes (GE Healthcare) using a TE 77 semidry blotting system (GE Healthcare) at 0.8 mA/cm for 1 h. Membranes were saturated for one hour with 8% milk powder in TBS (50 mM Tris-HCl, pH 7.6 and 150 mM NaCl) containing 0.1% Tween 20 (TBS/T). Blots were incubated overnight at 4°C with antibodies against vimentin (Abcam ab92547), LIM domain-binding protein 3 (Abcam ab154183), peroxiredoxin-5 (Abcam ab119712), apolipoprotein E (Abcam ab1906) and phospho Map4K4-ser801 (BioSource bs-5493R).

After washing three times in TBS/T, blots were incubated for one hour at room temperature with horseradish peroxidase-conjugated or alkaline phosphatase anti-mouse or anti-rabbit secondary antibody (Santa Cruz Biotechnology) in blocking buffer (TBS/T with 8% w/v milk powder). Immuno-detection was performed with ECL advance Western blotting detection kit (GE Healthcare). The protein bands were quantified using Total Lab (TL100) software (http://www.totallab.com). ATP synthase ß (Abcam ab14730) was used for normalization as it showed no significant change in the proteomics analysis.

### MAP4K4 ELISA assay

To test the amount of total MAP4K4 same amount of protein (100 μg) from each sample was used for the enzyme-linked immunosorbent assay (ELISA) assay that was performed according to manufacturer’s guidelines (MyBioSource MBS9317805).

### Statistical analysis

Statistical analysis was performed using Graph Pad Prism (release 4). Immunoblotting results were evaluated using non-paired Student´s t-test. Data are presented as means + standard deviation (SD). A p-value of less than 0.05 was considered to denote statistical significance. Three biological replicates were used in all experiments.

## Results

### Proteome alteration increases with the radiation dose

**Fig 1:**
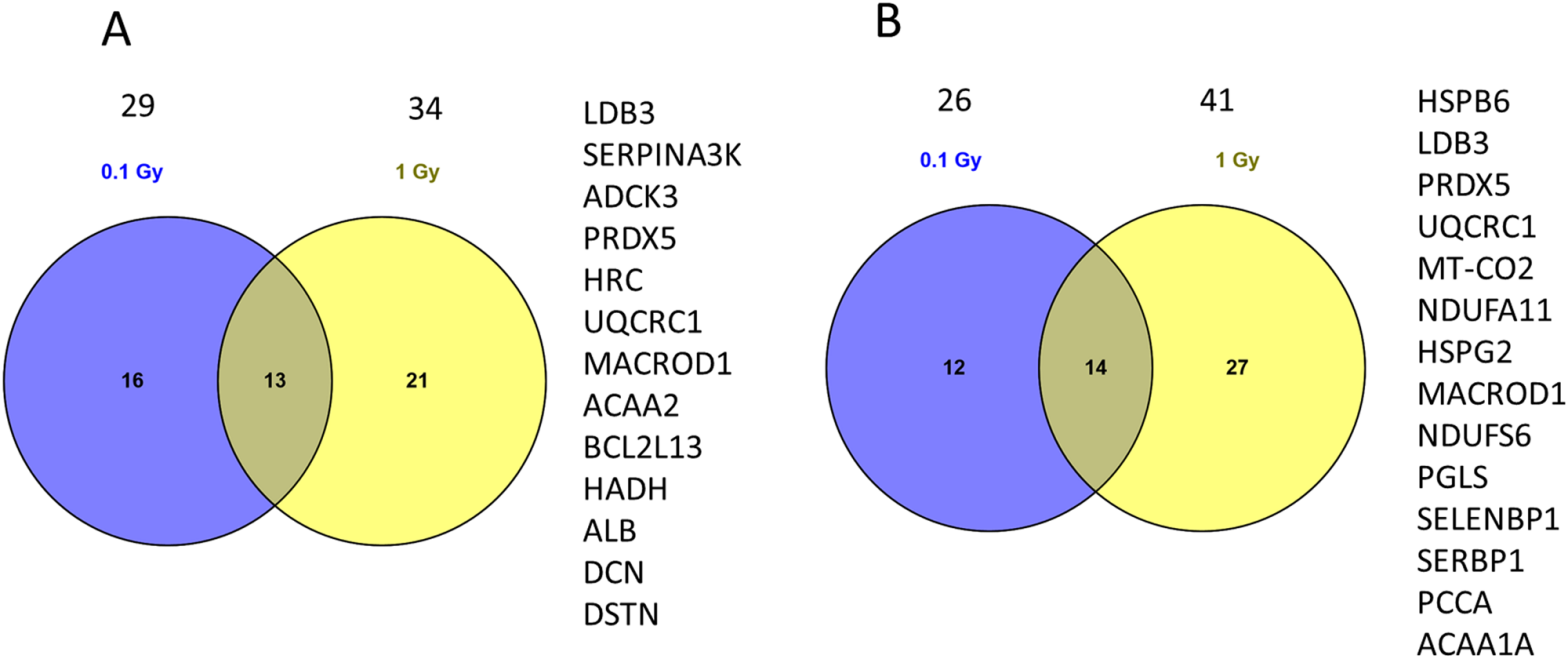
Venn diagrams showing the number of all and shared deregulated proteins at doses of 0.1 Gy and 1.0 Gy. (A) The numbers of deregulated proteins at 6 months and (B) 2 years are indicated above the circles. The list of shared proteins at these time points is shown on the right.

The proteomic analysis at 6 months using male offspring identified a total of 1196 proteins (682 quantified) of which 19 (1.6%) and 29 proteins (2.4%) were significantly deregulated at 0.02 Gy and 0.1 Gy, respectively (Tables A and B in S2 File). At 6 months, the number of identified proteins after 1.0 Gy was 1188 (644 quantified) of which 34 proteins (2.9%) were significantly deregulated (Table C in S2 File). The protein expression changes induced at the lowest dose (0.02 Gy) showed little overlap with those seen at the higher doses with only seven proteins shared with the deregulated proteins at the dose of 0.1 Gy or 1.0 Gy. However, thirteen significantly deregulated proteins were shared between 0.1 Gy and 1.0 Gy doses, including peroxiredoxin 5 (PRDX5), LIM domain-binding protein-3 (LDB3), and several mitochondrial proteins (Fig 1A).

As only a few proteome alterations were seen at the dose of 0.02 Gy, the effect of a slightly higher but still a low radiation dose (0.05 Gy) was investigated at a later time point (2 years). To estimate the effect of the gender in the radiation response female offspring was used at this time point. The proteomics analysis at 0.05 Gy and 0.1 Gy identified a total of 983 proteins (528 quantified) of which 11 (1.1%) and 26 proteins (2.7%) were significantly deregulated, respectively (Tables D and E in S2 File). At the 1.0 Gy dose 2 years post-irradiation, 988 proteins were identified (582 quantified), of which 41 proteins (4.2%) were significantly deregulated (Table F in S2 File). Again, the proteome response at the lowest dose was distinct from that of the higher doses with only six deregulated proteins shared with 0.1 Gy or 1.0 Gy. In contrast, the higher doses induced similar changes in the heart proteome. Fourteen significantly deregulated proteins were shared between these doses (Fig 1B). These included PRDX5, LDB3, heat shock protein HSPB6, and several mitochondrial proteins.

The proteome alteration at the highest dose (1.0 Gy) was investigated as a function of time. Ten deregulated proteins were found shared at 6 months and 2 years (Table 1), indicating a similar response in male and female mice. Members of mitochondrial complexes I and III, and hydroxyacyl-Coenzyme A dehydrogenase (HADH), involved in acetyl-CoA pathway, were found upregulated. Similarly, HSBP6, PRDX5, and LDB3 were upregulated whereas the structural protein vimentin (VIM) and the lipid translocator apolipoprotein E (APOE) were found downregulated (Table 1). With the exception of transgelin 2 (TAGLN2), an actin binding protein and a marker of differentiated smooth muscle [22], all shared proteins showed a similar direction of deregulation at the two time points. All differentially regulated proteins showed a greater fold change in expression at 2 years than at 6 months, suggesting a progressive proteome alteration.

**Table 1:** Significantly deregulated proteins after 1.0 Gy *in-utero* dose common to time points of 6 months and 2 years.

Protein name	Fold change		Function
	6 m	2 y	
Heat shock protein, alpha-crystallin-related, B6	2.3	3.7	Cardiac apoptosis
LIM-domain binding protein 3	2.2	2.4	Developmentally regulated in cardiac muscle
Peroxiredoxin 5	1.8	2.0	Response to mitochondrial oxidative stress
Hydroxyacyl-Coenzyme A dehydrogenase	1.6	1.7	Acetyl-CoA pathway
MACRO domain containing 1	1.5	1.7	Ribose deacetylase
Ubiquinol-cytochrome c reductase core protein 1	1.4	2.0	Mitochondrial respiratory Complex III
NADH dehydrogenase (ubiquinone) flavoprotein 2	1.3	1.4	Mitochondrial respiratory Complex I
Transgelin 2	0.7	1.4	Marker of differentiated smooth muscle
Vimentin	0.7	0.6	Structural constituent
Apolipoprotein E	0.6	0.4	Lipid transportation

Taken together, these data indicate a dose-dependent increase in the number of deregulated proteins at both time points. The proteome response at the lowest doses used here (0.02 Gy, 0.05 Gy) showed little similarity with that at the higher doses. Nevertheless, the protein LDB3 that is essential for the structure of sarcomeres was found upregulated at all doses and time points.

### Deregulated proteins form mitochondrial, acute phase, and structural protein clusters

Proteins, the expression of which was altered at the lowest radiation doses, i.e. 0.02 Gy and 0.05 Gy, (6 months and 2 years, respectively) could not be clustered to form an interactive network using STRING analysis. This suggested that no particular biological pathway was significantly affected at these doses.

**Fig 2:**
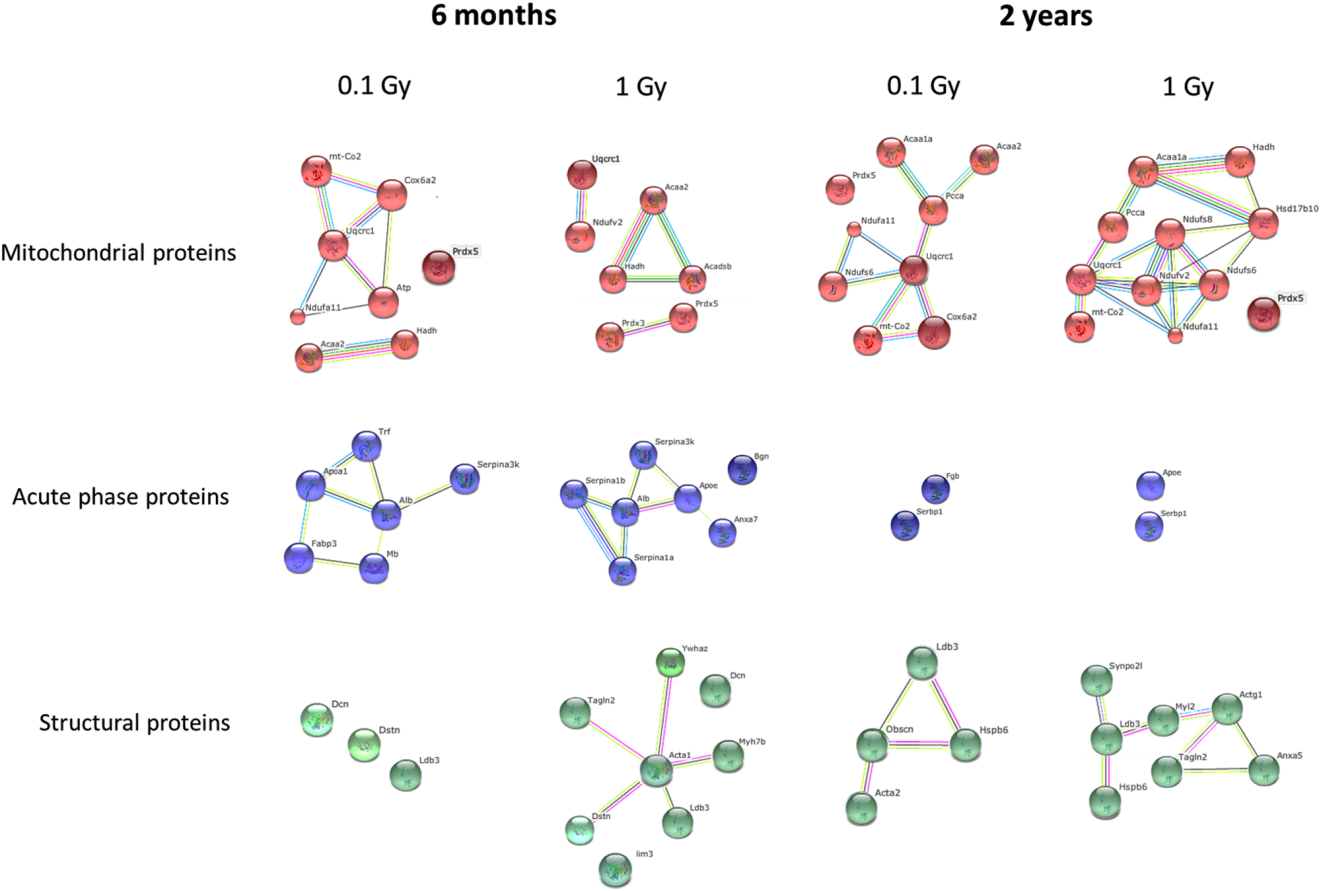
STRING protein networks of significantly changed proteins at prenatal (E11) radiation exposure of 0.1 Gy and 1.0 Gy. Common networks between 6-month- and 2-year-time points are shown. Mitochondrial proteins, acute phase proteins and structural proteins represent the major protein classes of proteins affected by the pre-natal irradiation.

Significantly deregulated proteins at doses of 0.1 Gy or 1.0 Gy could be clustered in categories as follows: mitochondrial proteins, acute phase response and structural proteins (cytoskeleton) (Fig 2). In most cases, the number of proteins in a particular network increased with dose at both time points, supporting a dose-dependent increase of proteome alterations that was also suggested by the proteomics analysis. In case of mitochondrial proteins, several members of the respiratory complexes I, III and IV showed persistent upregulation, ubiquinol-cytochrome c reductase (UQCRC1) being a central member of all networks. Although the networks of acute phase and structural proteins were represented as deregulated at both time points, the members of these networks changed in a time-dependent manner. For example, a transient deregulation was seen in the serpine family of proteins at 1.0 Gy at 6 months but not at 2 years. Some of these changes may also be due to a gender-specific alteration of the cardiac proteome.

### Immunoblotting confirms the results of the proteomic analysis

**Fig 3:**
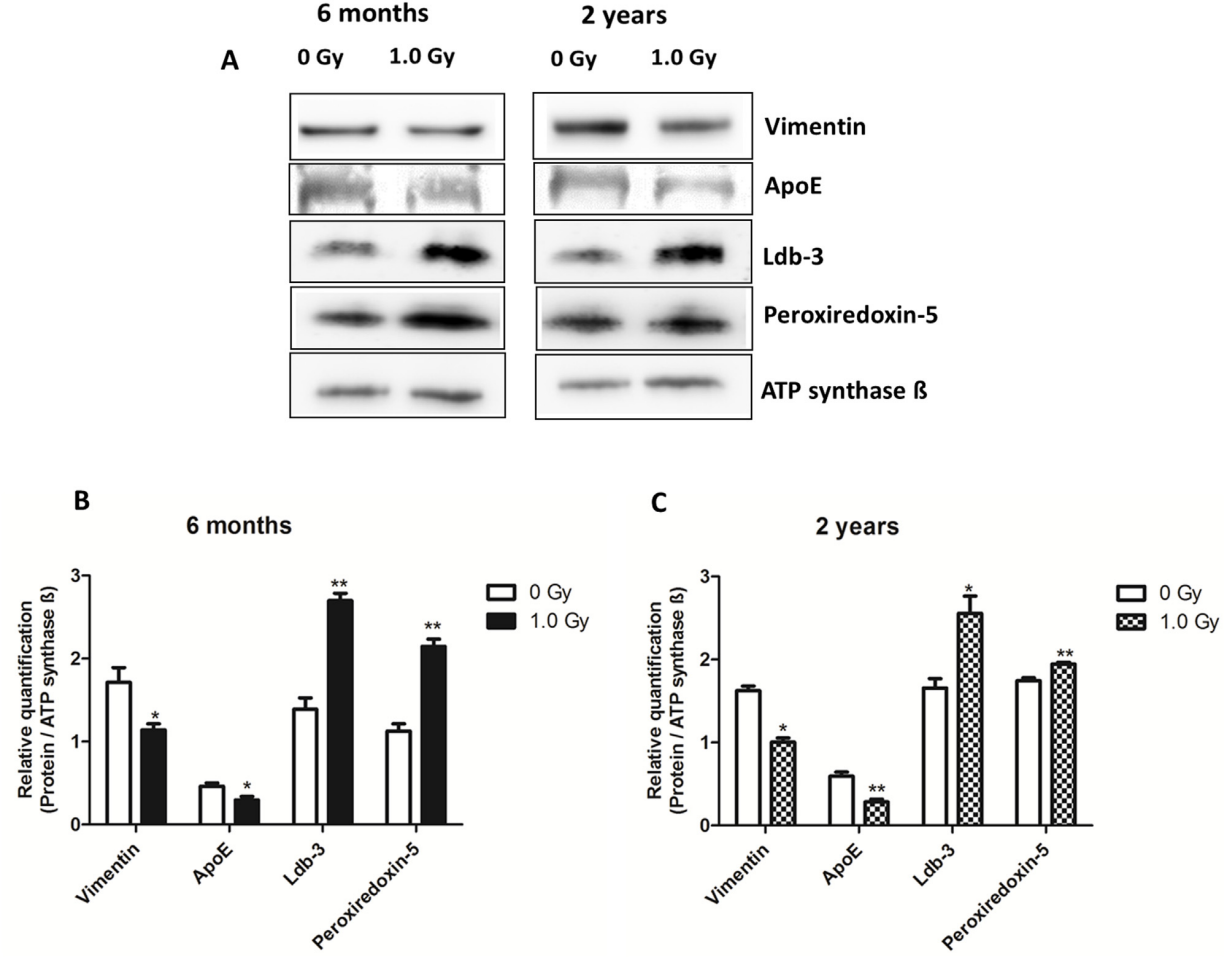
Immunoblot validation of proteomics data at 1.0 Gy using antibodies against vimentin (VIM), apolipoprotein E (APOE), LIM domain-binding protein (LDB3), and peroxiredoxin 5 (PRDX5). (A) The immunoblot images of VIM, APOE (6 months), PRDX5, and LDB3 are shown. The bar charts at 6 months (B) and 2 years (C) represent the average ratios with standard deviation (SD) of relative protein expression in control and 1.0 Gy irradiated samples after background correction and normalization to ATP synthase ß. (unpaired Student´s t-test; *p ≤0.05; **p≤0.01; n = 3).

In order to validate the proteomics data, the expression of proteins representing the biological categories of cellular structure, metabolism or oxidative stress was investigated by immunoblotting (Fig 3). In agreement with the proteomics data, immunoblotting showed increase in the amount of LDB3 and PRDX5 whereas a decrease in the level of structural protein vimentin and lipid transporter APOE was observed after the 1.0 Gy dose at both time points (Figs A-D in S1 File).

### Expressions of the total MAP4K4 and phospho-MAP4K4 are decreased after 1.0 Gy

**Fig 4:**
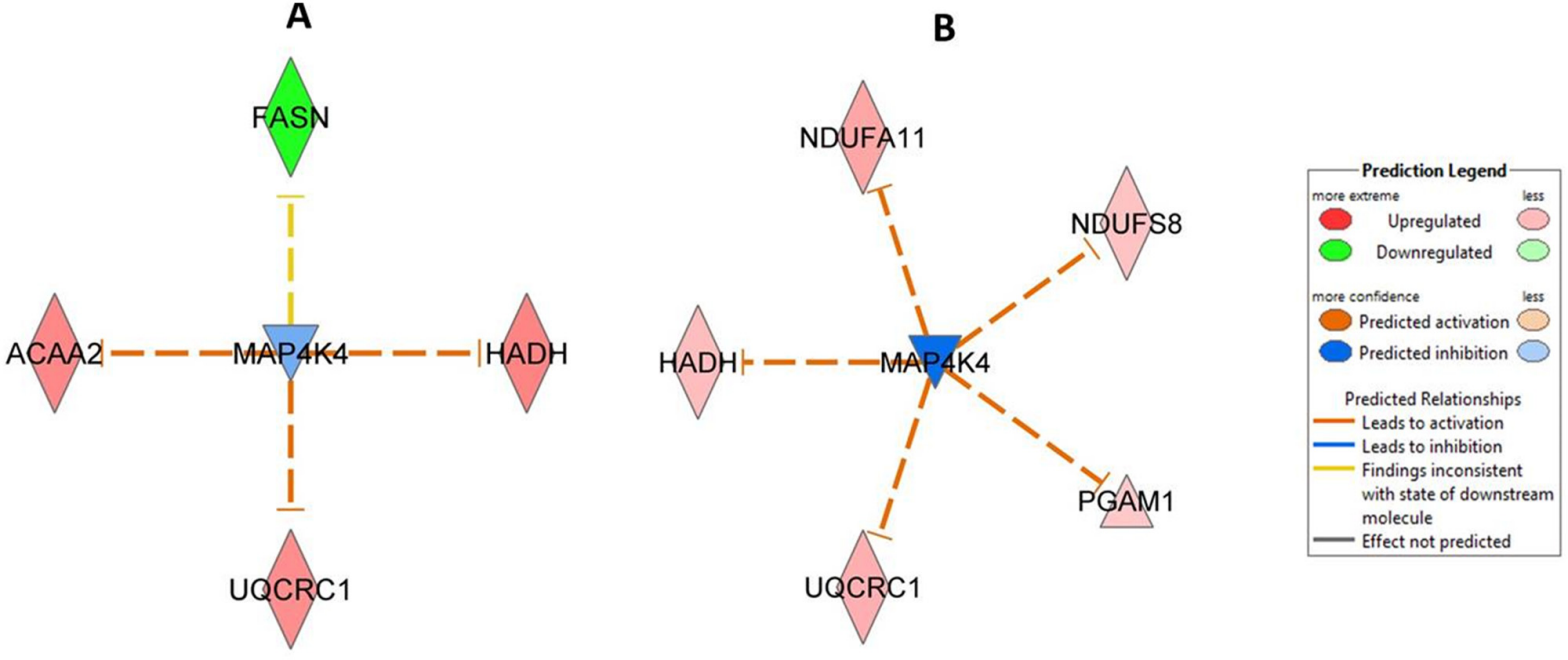
Ingenuity Pathway Analysis networks showing predicted inhibition of protein kinase MAP4K4. (A) Predicted networks after *in utero* irradiation at 1.0 Gy, 6 months and (B) 1.0 Gy, 2 year are shown. Three biological replicates were used in all experiments. ACAA2, acetyl-Coenzyme A acyltransferase 2; FASN, fatty acid synthase; HADH, hydroxyacyl-Coenzyme A dehydrogenase; UQCRC1, ubiquinol-cytochrome c reductase core protein 1; NDUFA11, NADH dehydrogenase (ubiquinone) 1 alpha subcomplex 11; NDUFS8, NADH dehydrogenase (ubiquinone) Fe-S protein 8; PGAM1, phosphoglycerate mutase 1; MAP4K4, Mitogen-activated protein kinase kinase kinase kinase 4.

Ingenuity Pathway Analysis (IPA) predicted the inhibition of mitogen-activated protein kinase kinase kinase kinase 4 (MAP4K4) at 6 and 24 months after a dose of 1.0 Gy (Fig 4) but not at lower doses.

**Fig 5:**
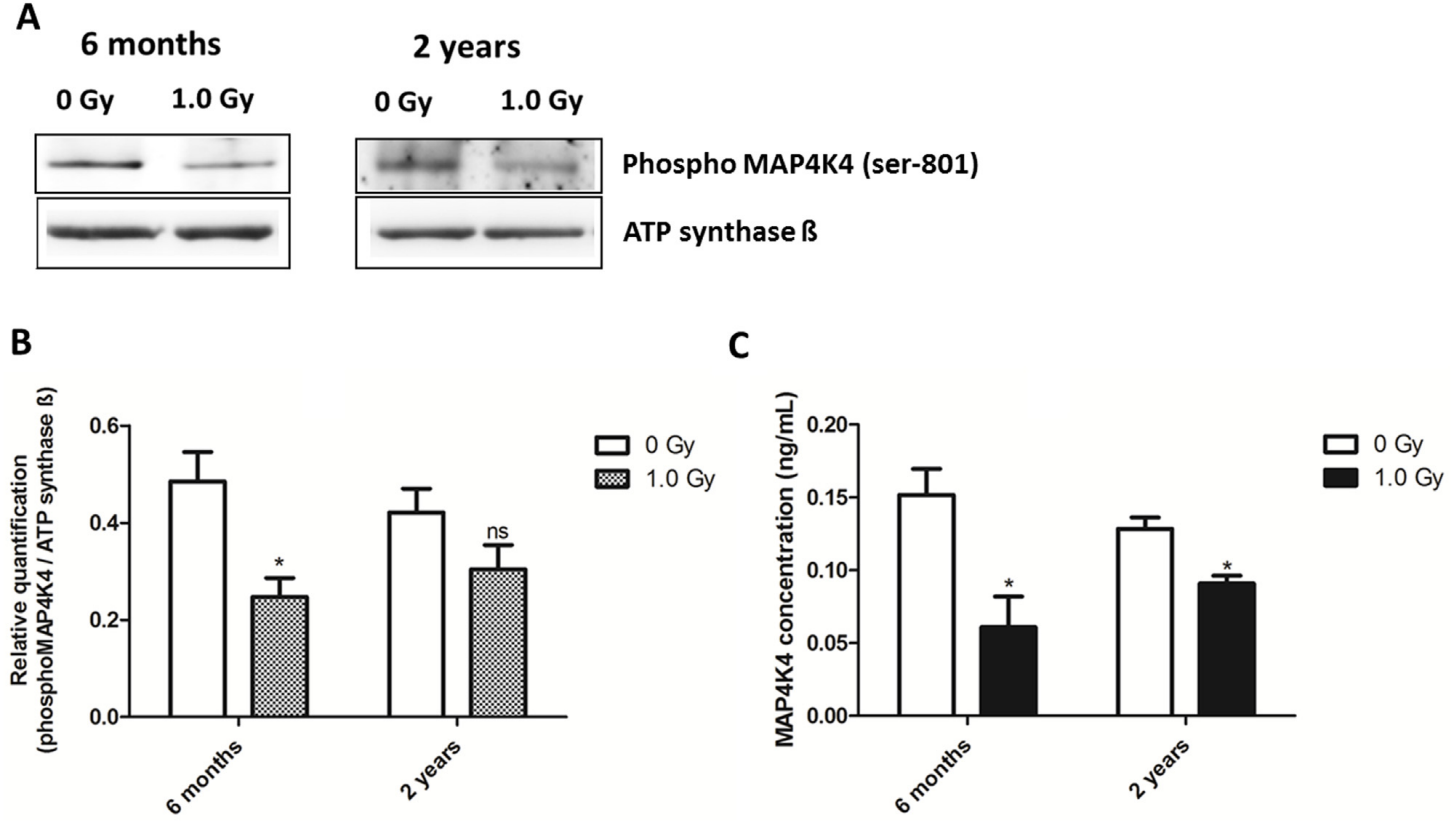
Characterization of MAP4K4 in the control and 1.0 Gy-irradiated mouse heart. (A) The immunoblot images of phospho-MAP4K4 (Ser-801) in the 1.0 Gy irradiated hearts compared to the controls at 6 months and 2 years is shown. (B) Columns represent the average ratios with standard deviation (SD) of relative protein expression in control and 1.0 Gy irradiated samples after background correction and normalization to ATP synthase ß (unpaired Student´s t-test; *p ≤0.05; ns, non-significant; n = 3). (C) The total amount of MAP4K4 measured using ELISA in control and 1.0 Gy irradiated heart tissue shows significant radiation-induced decrease at 6 months and 2 years (unpaired Student´s *t*-test; *p ≤0.05; ns, non-significant; n = 3).

The relative quantification of the level of phosphorylated (active) MAP4K4 (Ser-801) using immunoblotting showed a significant decrease at 6 months (Fig 5A and Fig D in S1 File). At 2 years, the significance of downregulation was not reached (p = 0.1714) (Fig 5A).

To confirm the predicted inhibition of MAP4K4, the total amount was measured using the control and 1.0 Gy irradiated cardiac tissue. A significant decrease in the level of total kinase expression was observed at both time points in irradiated hearts (Fig 5B).

## Discussion

There is a considerable lack of data concerning late effects of radiation-induced damage in the prenatal period. This study is to our knowledge the first to investigate persistent molecular changes in the murine heart after *in-utero* exposure to low and moderate doses of ionizing radiation. The doses used here, ranging from 0.02 to 1.0 Gy, are comparable to those found in medical diagnostic situations, atomic bombings and occupationally exposed populations [23,24].

We show here that many mitochondrial proteins show differential expression after prenatal irradiation (E11) already at the dose of 0.1 Gy. Recent data strongly suggest that the switch to aerobic metabolism in the murine embryonic heart occurs around embryonic day E11.5 [25], close to the time point of irradiation in this study. It has been shown that at E9.5 mitochondrial electron transfer chain (ETC) activity and oxidative phosphorylation (OXPHOS) are not coupled, even though the complexes are present. At E11.5, mitochondria appear functionally more mature, ETC activity and OXPHOS are coupled and respond to ETC inhibitors [25]. The time window of OXPHOS activation may be especially susceptible to radiation-induced mitochondrial impairment. In this study, many members of respiratory Complexes I and IV are found differentially regulated after prenatal exposure, this being in agreement with the data following neonatal irradiation with same doses as used here [21]. In addition, upregulation of Complex III proteins is seen in this study. Our previous data on locally irradiated adult (8 weeks) mouse hearts (2 Gy) also highlight mitochondrial respiratory complexes as radiation targets in the heart but mice irradiated in the adulthood show downregulation, not upregulation, of the complex proteins [26]. This was connected with increased reactive oxygen species formation and protein oxidation, even 40 weeks after the radiation exposure [27]. The prenatal irradiation used here does not increase protein oxidation in the heart (data not shown) although the persistent upregulation of PRDX5 is indicative of long-term oxidative stress [28,29]. The upregulation of respiratory chain proteins may indicate a protective response to the early transient radiation insult [30].

A second group of significantly deregulated proteins found in this study are the heat shock proteins. Similar to mitochondrial proteins, the marked upregulation of heat shock protein B6 (HSPB6, formerly heat shock protein 20) that is observed at the 1.0 Gy dose already after 6 months and at all doses after 2 years, may indicate a response to persistent radiation-induced damage. HSPB6 plays a key role in protection against apoptosis, remodeling, and ischemia/reperfusion injury [31,32]. It has been implicated in modulation of cardiac contractility through sarcoplasmic reticulum calcium cycling [33].

Also involved in the cardiac contractility is the protein LDB3, also known as Cypher or Z-band alternatively spliced PDZ-motif (ZASP). It is the only protein found upregulated at all doses and time points. It stabilizes the cardiac sarcomere during contraction, through interactions with actin [34]. Mutations in LDB3 cause several forms of heart disease including dilated cardiomyopathy [35,36]. Another Z-disc protein, SYNPO2L, highly expressed in the mouse embryonic heart [37] and essential for heart function [38], is found almost twofold upregulated two years after the prenatal radiation dose of 1.0 Gy.

A transient deregulation (both up- and downregulation) at six months is seen of many members of the serpin family, especially at 1.0 Gy. The serpin family comprises a structurally similar, yet functionally diverse, set of proteins. Named originally for their function as serine proteinase inhibitors, many of its members are not inhibitors but rather chaperones, involved in cellular storage and transport [39]. An almost twofold upregulation is found at 1.0 Gy for the expression of SERPINA1A, alpha-1-antitrypsin orthologue, the deletion of which is embryonically lethal [40]. Alpha-1-antitrypsin expression has been associated with atherosclerosis progression in human [41].

An alteration of the cardiac cytoskeleton is suggested already at 0.1 Gy. The proteins from the structural component category such as annexin 5, transgelin (2 years), obscurin, and different forms of actin and myosin show increased expression. Obscurin plays a role in the formation of new sarcomeres during myofibril assembly [42] and mutations in this protein have been associated with dilated [43] and hypertrophic cardiomyopathy [44]. In contrast, downregulation of the vimentin expression was observed here, in accordance with our previous studies using adult mice [26,27].

APOE has an important function in the heart by facilitating the transport of high density lipoproteins (HDL) and low density lipoproteins (LDL) across the plasma membrane [45]. The downregulation of APOE observed at both time points (1.0 Gy) may suggest increased lipid accumulation [46] as previously shown at high doses of ionizing radiation to the heart [47].

This study shows that *in-utero* irradiation (1.0 Gy) decreases the expression of total and active form of MAP4K4 by reducing the phosphorylation of serine-801. MAP4K4, a serine/threonine protein kinase [48], is essential in embryonic mesoderm formation leading to the origin of the cardiovascular system [49]. The deletion of the corresponding gene is lethal as the k.o. mice die between E9.5 and E10.5. (Xue, Wang et al. 2001). Little is known of its function in the heart but several isoforms of this enzyme were identified recently in the rat cardiac kinome [50]. Its expression was higher in neonatal ventricular myocytes compared to the adult ones. MAP4K4 may play a role in the response to environmental stress and inflammation as silencing it in macrophages *in vivo* protected mice from lipopolysaccharide-induced lethality by inhibiting TNF-alpha and interleukin-1beta production [51]. The reduced amount of total and active form of MAP4K4 seen in this study may be protective against cardiac inflammation but more research is needed to clarify this.

As we find no great differences in the heart proteome responses of male and female mice, the gender-specific responses may be characteristic only to mice irradiated at a mature age. This is in agreement with our previous studies using both male and female mice that were irradiated early in life at postnatal day 10. These studies showed little difference in the hippocampal proteome after low and moderate doses of ionizing radiation [17,52].

## Conclusions

This study suggests that biological pathways important at the time of irradiation (E11) are still found altered years after the initial insult. Such pathways include initiation of mitochondrial respiration and activation of MAP kinases in the mouse embryonic heart. The proteomic response after prenatal irradiation is different from that observed after radiation exposure at the adult age but bears resemblance to that found after radiation exposure at the early postnatal phase. Several structural proteins found dysregulated in this study are involved in the sarcomere formation and contractility. Both of these processes are rapidly developing at the time of the radiation exposure.

## Supporting Information

S1 File Supplementary Figures. Fig A: Images from immunoblotting of APOE and ATP synthase ß in control and 1.0 Gy irradiated heart lysates at 6 months and 2 years. Fig B: Images from immunoblotting of PRDX5 and ATP synthase ß in control and 1.0 Gy irradiated heart lysates at 6 months and 2 years. Fig C: Images from immunoblotting of LDB3 and ATP synthase ß in control and 1.0 Gy irradiated heart lysates at 6 months and 2 years. Fig D: Images from immunoblotting of phospho‐MAP4K4 (Ser‐801), vimentin and ATP synthase ß in control and 1.0 Gy irradiated heart lysates at 6 months and 2 years.
(PDF)

S2 File Supplementary tables. Table A: Significantly deregulated proteins at 0.02 Gy, 6 months. Table B: Significantly deregulated proteins at 0.1 Gy, 6 months. Table C: Significantly deregulated proteins at 1.0 Gy dose, 6 months. Table D: Significantly deregulated proteins list at 0.05 Gy, 2 years. Table E: Significantly deregulated proteins at 0.1 Gy, 2 years. Table F: Significantly deregulated proteins at 1.0 Gy, 2 years.
(PDF)

## Abbreviations

Gy: Gray
IPA: Ingenuity Pathway Analysis
H/L: heavy to light ratio
ICPL: isotope coded protein label
LC-MS: liquid chromatography mass spectrometry
