# Supplementary material for: In-Utero Low-Dose Irradiation Leads to Persistent Alterations in the Mouse Heart Proteome
Source: PLoS One. 2016 Jun 8;11(6):e0156952. doi: 10.1371/journal.pone.0156952 (PMC4898684; doi:10.1371/journal.pone.0156952)

Supplementary figures

Figure A. Images from immunoblotting of APOE and ATP synthase ß in control and 1.0 Gy irradiated heart lysates at 6 months and 2 years.

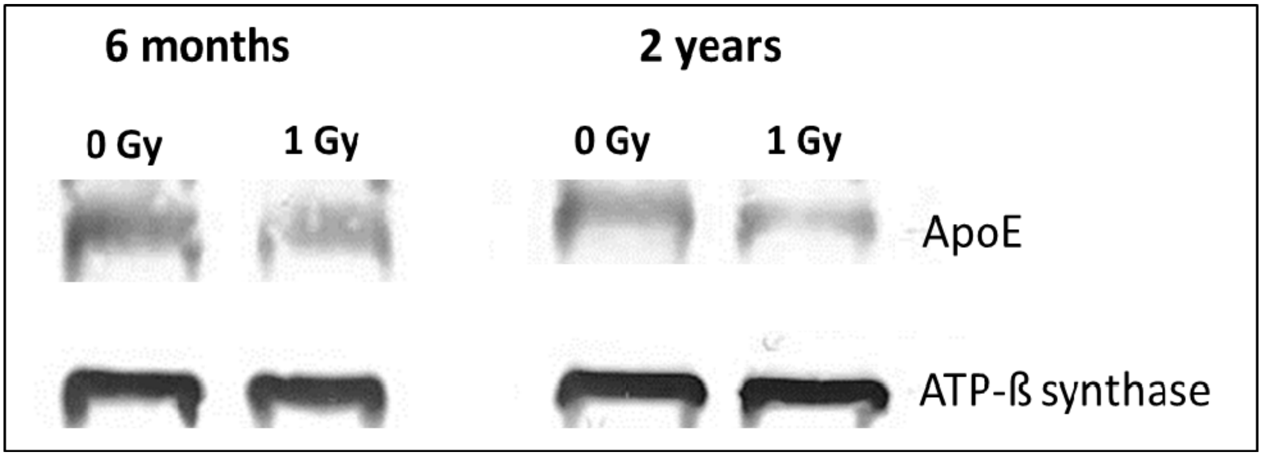

Figure B. Images from immunoblotting of PRDX5 and ATP synthase ß in control and 1.0 Gy irradiated heart lysates at 6 months and 2 years.

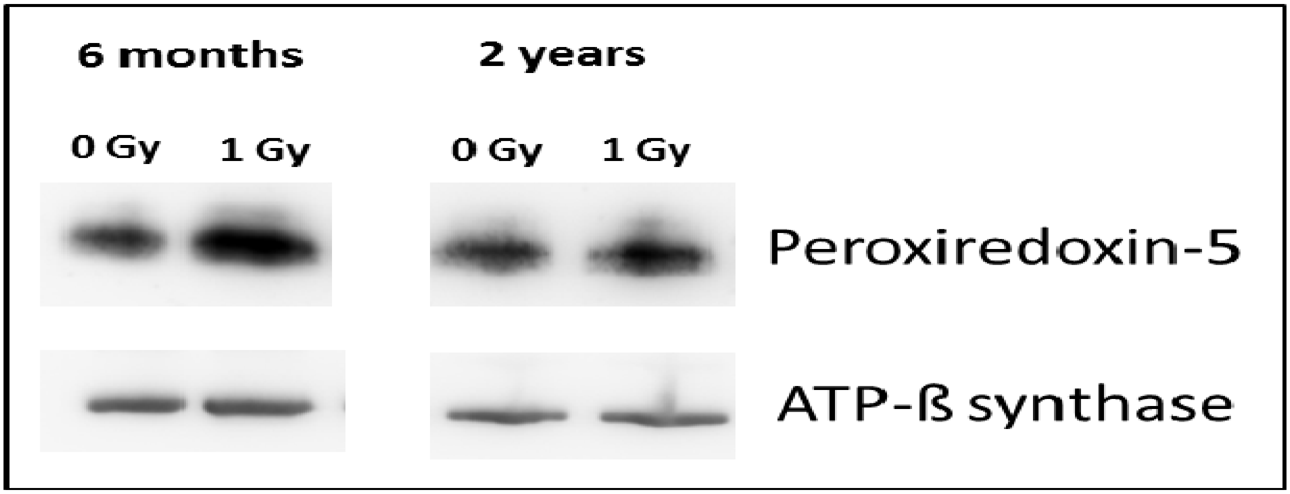

Figure C. Images from immunoblotting of LDB3 and ATP synthase ß in control and 1.0 Gy irradiated heart lysates at 6 months and 2 years.

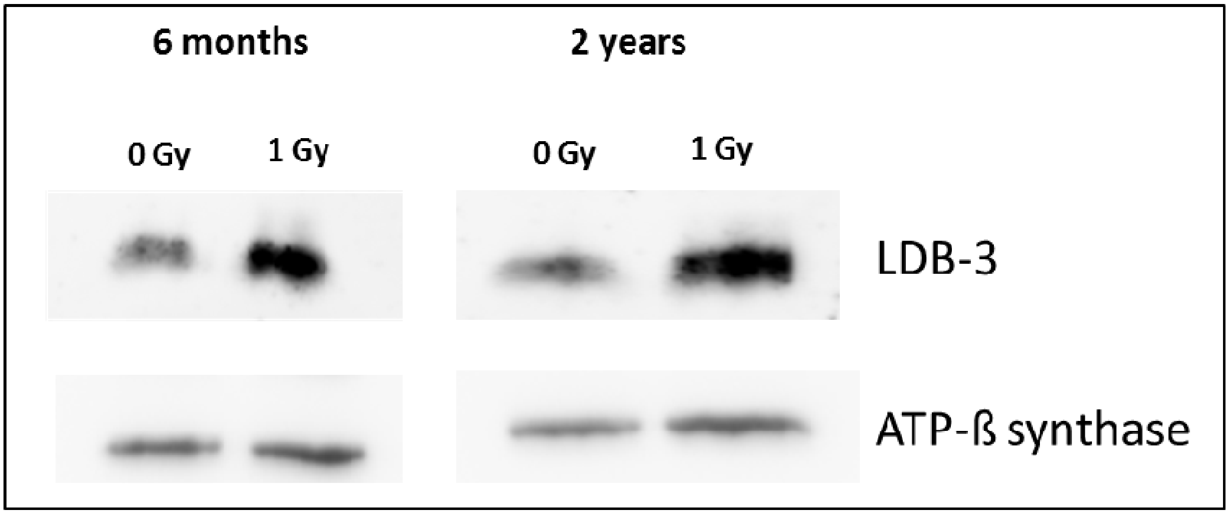

Figure D. Images from immunoblotting of phospho-MAP4K4 (Ser-801), vimentin and ATP synthase ß in control and 1.0 Gy irradiated heart lysates at 6 months and 2 years.

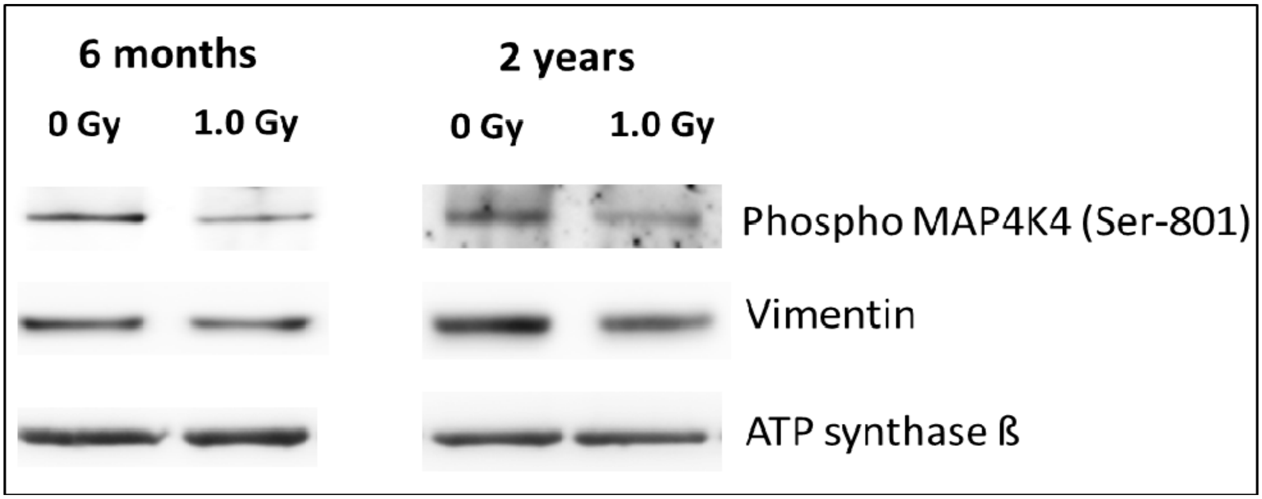

Supplement: S1 File — Fig A: Images from immunoblotting of APOE and ATP synthase ß in control and 1.0 Gy irradiated heart lysates at 6 months and 2 years. Fig B: Images from immunoblotting of PRDX5 and ATP synthase ß in control and 1.0 Gy irradiated heart lysates at 6 months and 2 years. Fig C: Images from immunoblotting of LDB3 and ATP synthase ß in control and 1.0 Gy irradiated heart lysates at 6 months and 2 years. Fig D: Images from immunoblotting of phospho‐MAP4K4 (Ser‐801), vimentin and ATP synthase ß in control and 1.0 Gy irradiated heart lysates at 6 months and 2 years. (PDF) [file pone.0156952.s001.pdf]
