# Supplementary material for: In-Utero Low-Dose Irradiation Leads to Persistent Alterations in the Mouse Heart Proteome
Source: PLoS One. 2016 Jun 8;11(6):e0156952. doi: 10.1371/journal.pone.0156952 (PMC4898684; doi:10.1371/journal.pone.0156952)
Supplement: S2 File — Table A: Significantly deregulated proteins at 0.02 Gy, 6 months. Table B: Significantly deregulated proteins at 0.1 Gy, 6 months. Table C: Significantly deregulated proteins at 1.0 Gy dose, 6 months. Table D: Significantly deregulated proteins list at 0.05 Gy, 2 years. Table E: Significantly deregulated proteins at 0.1 Gy, 2 years. Table F: Significantly deregulated proteins at 1.0 Gy, 2 years. (PDF) [file pone.0156952.s002.pdf]

Supplementary tables

Table A. Significantly deregulated proteins at 0.02 Gy, 6 months.

ENSEMBL ID	Symbol	Protein name	Unique Peptides	Fold changes	Variability [%]
ENSMUSP00000046497	ACAD12	Acyl-Coenzyme A dehydrogenase family, member 12	4	1,643	0,7
ENSMUSP00000022328	LDB3	LIM domain binding 3	4	1,524	4,1
ENSMUSP00000080584	MYH13	Myosin, heavy polypeptide 13, skeletal muscle	2	1,439	16,6
ENSMUSP00000033049	COX6A2	Cytochrome c oxidase, subunit VI a, polypeptide 2	2	1,377	3,1
ENSMUSP00000087192	BDH1	3-hydroxybutyrate dehydrogenase, type 1	8	0,761	6,6
ENSMUSP00000026576	TALDO1	Transaldolase 1	2	0,753	6,9
ENSMUSP00000125636	CYB5R3	Cytochrome b5 reductase 3	3	0,749	5,2
ENSMUSP00000035158	TRF	Transferrin	10	0,746	3,2
ENSMUSP00000037206	FKBP	FK506 binding protein 1a	4	0,732	17,9
ENSMUSP00000121961	RPS5	Ribosomal protein S5	6	0,732	13,0
ENSMUSP00000099461	DSTN	Destrin	3	0,730	20,5
ENSMUSP00000100924	DCN	Decorin	6	0,719	11,6
ENSMUSP00000082132	SERPINA1A	Serine peptidase inhibitor, clade A, member 1A	4	0,697	10,8
ENSMUSP00000034588	APOA1	Apolipoprotein A-I	8	0,689	23,5
ENSMUSP00000033741	BGN	Biglycan	5	0,676	16,4
ENSMUSP00000104108	RTN2	Reticulon 2	2	0,662	12,2
ENSMUSP00000031314	ALB	Albumin	11	0,632	10,9
ENSMUSP00000034053	PDLIM3	PDZ and LIM domain 3	2	0,617	13,5
ENSMUSP00000130377	COL1A2	Collagen, type I, alpha 2	2	0,501	15,3

Table B. Significantly deregulated proteins at 0.1 Gy, 6 months.

ENSEMBL ID	Symbol	Protein	Unique Peptides	Fold changes	Variability [%]
ENSMUSP00000022328	LDB3	LIM domain binding 3	4	2,300	10,1
ENSMUSP00000042095	SERPINA3K	Serine (or cysteine) peptidase inhibitor	3	2,260	26,9
ENSMUSP00000033049	COX6A2	Cytochrome c oxidase, subunit VI a,	2	2,095	12,8
ENSMUSP00000027766	ADCK3	AarF domain containing kinase 3	4	1,867	4,2
ENSMUSP00000134521	PRDX5	Peroxiredoxin 5	2	1,862	11,1
ENSMUSP00000001242	D10JHU	DNA segment, Chr 10, Johns Hopkins University	3	1,781	12,1
ENSMUSP00000043501	HRC	Histidine rich calcium binding protein	12	1,756	10,3
ENSMUSP00000080994	MT-CO2	Mitochondrially encoded cytochrome c oxidase II	4	1,604	17,6
ENSMUSP00000026743	UQCRC1	Ubiquinol-cytochrome c reductase core protein 1	17	1,574	17,5
ENSMUSP00000125939	PCCB	Propionyl Coenzyme A carboxylase, beta polypeptide	7	1,546	6,5
ENSMUSP00000039507	MACROD1	MACRO domain containing 1	8	1,541	17,8
ENSMUSP00000037348	ACAA2	Acetyl-Coenzyme A acyltransferase 2	8	1,439	5,7
ENSMUSP00000001304	CKB	Creatine kinase	6	1,428	18,7
ENSMUSP00000032887	COQ7	Demethyl-Q 7	4	1,419	26,6
ENSMUSP00000002452	NDUFA11	NADH dehydrogenase (ubiquinone) 1 alpha subcomplex 11	3	1,385	11,2
ENSMUSP00000019037	MB	Myoglobin	2	1,381	12,1
ENSMUSP00000009256	BCL2L13	BCL2-like 13 (apoptosis facilitator)	2	1,365	6,6
ENSMUSP00000029610	HADH	Hydroxyacyl-Coenzyme A dehydrogenase	2	1,365	16,0
ENSMUSP00000122103	SLC25A12	Solute carrier family 25 (mitochondrial carrier, Aralar)	14	1,349	20,6
ENSMUSP00000070709	FABP3	Fatty acid binding protein 3, muscle and heart	3	1,348	7,3
ENSMUSP00000123100	ATP5C1	ATP synthase, H+ transporting, mitochondrial F1	3	0,756	15,5
ENSMUSP00000051570	SYNPO2	Synaptopodin 2	4	0,743	10,0
ENSMUSP00000026625	CLYBL	Citrate lyase beta	5	0,732	6,4
ENSMUSP00000035158	TRF	Transferrin	10	0,728	4,3
ENSMUSP00000031314	ALB	Albumin	11	0,701	3,3
ENSMUSP00000100924	DCN	Decorin	6	0,695	18,4

**ENSMUSP00000034588**	**APOA1**	**Apolipoprotein A-I**	**8**	**0,689**	**4,2**
**ENSMUSP00000099461**	**DSTN**	**Destrin**	**3**	**0,685**	**9,7**
**ENSMUSP00000106602**	**YWHAQ**	**Tyrosine 3-monooxygenase**	**2**	**0,612**	**15,8**

Table C. Significantly deregulated proteins at 1.0 Gy dose, 6 months.

ENSEMBL ID	Symbol	Protein	Unique Peptides	Fold changes	Variability [%]
ENSMUSP00000039172	HSPB6	Heat shock protein, alpha-crystallin-related, B6	5	2,344	25,3
ENSMUSP00000022328	LDB3	LIM domain binding 3	4	2,197	22,2
ENSMUSP00000084509	FN1	Fibronectin 1	9	2,159	30,6
ENSMUSP00000034453	ACTA1	Actin, alpha 1, skeletal muscle	2	2,104	20,7
ENSMUSP00000082132	SERPINA1A	Serine (or cysteine) peptidase inhibitor, clade A	4	1,946	4,4
ENSMUSP00000042095	SERPINA3K	Serine (or cysteine) peptidase inhibitor, clade A, member 3K	3	1,944	21,4
ENSMUSP00000134521	PRDX5	Peroxiredoxin 5	1	1,790	15,1
ENSMUSP00000043501	HRC	Histidine rich calcium binding protein	15	1,659	13,4
ENSMUSP00000029610	HADH	Hydroxyacyl-Coenzyme A dehydrogenase	2	1,583	4,4
ENSMUSP00000027766	ADCK3	ArF domain containing kinase 3	4	1,551	3,7
ENSMUSP00000037348	ACAA2	Acetyl-Coenzyme A acyltransferase 2	9	1,533	12,2
ENSMUSP00000039507	MACROD1	MACRO domain containing 1	7	1,500	18,6
ENSMUSP00000026743	UQCRC1	Ubiquinol-cytochrome c reductase core protein 1	15	1,456	6,1
ENSMUSP00000031038	OCAID1	OCIA domain containing 1	2	1,428	6,6
ENSMUSP00000121557	NDUFV2	NADH dehydrogenase (ubiquinone) flavoprotein 2	7	1,355	23,2
ENSMUSP00000009256	BCL2L13	BCL2-like 13 (apoptosis facilitator)	2	1,349	26,4
ENSMUSP00000098405	ANXA7	Annexin A7	5	1,343	30,2
ENSMUSP00000025961	PRDX3	Peroxiredoxin 3	3	1,339	27,4
ENSMUSP00000090672	MYH7B	Myosin, heavy chain 7B, cardiac muscle, beta	2	1,338	29,1
ENSMUSP00000093101	SERPINA1B	Serine (or cysteine) preptidase inhibitor, clade A, member 1B	3	1,338	0,2
ENSMUSP00000104830	UBE2V1	Ubiquitin-conjugating enzyme E2 variant 1	3	0,768	2,7
ENSMUSP00000022894	YWHAZ	Tyrosine 3-monooxygenase/tryptophan 5-monooxygenase activation protein	2	0,767	3,5
ENSMUSP00000129233	VIM	Vimentin	24	0,755	10,6
ENSMUSP00000031314	ALB	Albumin	11	0,753	7,2
ENSMUSP00000088372	GM10166	Predicted pseudogene	6	0,736	4,5

ENSMUSP00000015829	ACADSB	Acyl-Coenzyme A dehydrogenase, short/branched chain	2	0,734	3,2
ENSMUSP00000033741	BGN	Biglycan	3	0,721	1,0
ENSMUSP00000106859	TAGLN2	Transgelin 2	4	0,711	17,5
ENSMUSP00000099461	DSTN	Destrin	4	0,701	7,7
ENSMUSP00000100924	DCN	Decorin	5	0,686	13,4
ENSMUSP00000003066	APOE	Apolipoprotein E	5	0,644	17,9
ENSMUSP00000034053	PDLIM3	PDZ and LIM domain 3	2	0,636	16,1
ENSMUSP00000125164	GPD1	Glycerol-3-phosphate dehydrogenase 1 (soluble)	3	0,456	27,7
ENSMUSP00000052872	FASN	Fatty acid synthase	19	0,297	6,6

Table D. Significantly deregulated proteins list at 0.05 Gy, 2 years.

ENSEMBL ID	Symbol	Protein	Unique Peptides	Fold changes	Variability [%]
ENSMUSP00000099938	MDH1	Malate dehydrogenase 1	2	2,248	27,4
ENSMUSP00000039172	HSPB6	Heat shock protein, alpha-crystallin-related, B6	5	2,168	29,2
ENSMUSP00000028944	ACSS1	Acyl-CoA synthetase short-chain family member 1	11	1,503	4,5
ENSMUSP00000131316	HSPG2	Perlecan (heparan sulfate proteoglycan 2)	53	1,379	26,3
ENSMUSP00000022328	LDB3	LIM domain binding 3	5	1,368	7,7
ENSMUSP00000037341	NDUFB7	NADH dehydrogenase (ubiquinone) 1 beta subcomplex, 7	6	1,353	21,6
ENSMUSP00000034264	PGLS	6-phosphogluconolactonase	9	1,330	29,1
ENSMUSP00000101898	UBE2D3	Ubiquitin-conjugating enzyme E2D	2	0,737	5,0
ENSMUSP00000117800	GRHPR	Glyoxylate reductase/hydroxypyruvate reductase	4	0,722	23,8
ENSMUSP00000098314	MECP2	Methyl CpG binding protein 2	3	0,719	30,4
ENSMUSP00000135439	ACAA1A	Acetyl-Coenzyme A acyltransferase 1A	4	0,558	7,4

Table E. Significantly deregulated proteins at 0.1 Gy, 2 years.

ENSEMBL ID	Symbol	Protein	Unique Peptides	Fold changes	Variability [%]
ENSMUSP00000039172	HSPB6	Heat shock protein, alpha-crystallin-related, B6	5	2,623	28,4
ENSMUSP00000022328	LDB3	LIM domain binding 3	5	2,043	25,3
ENSMUSP00000033049	COX6A2	Cytochrome c oxidase, subunit VI a, polypeptide 2	2	1,896	14,2
ENSMUSP00000134521	PRDX5	Peroxiredoxin 5	2	1,861	1,3
ENSMUSP00000026743	UQCRC1	Ubiquinol-cytochrome c reductase	15	1,823	12,9
ENSMUSP00000080994	MT-CO2	Mitochondrially encoded cytochrome c oxidase II	4	1,811	2,4
ENSMUSP00000048218	ACTA2	Actin, alpha 2, smooth muscle, aorta	6	1,792	21,3
ENSMUSP00000002452	NDUFA11	NADH dehydrogenase (ubiquinone) 1 alpha subcomplex	3	1,714	28,4
ENSMUSP00000131316	HSPG2	Perlecan (heparan sulfate proteoglycan 2)	53	1,636	29,9
ENSMUSP00000039507	MACROD1	MACRO domain containing 1	6	1,621	12,0
ENSMUSP00000049737	OBSCN	Obscurin, cytoskeletal calmodulin and titin-interacting RhoGEF	13	1,590	5,3
ENSMUSP00000114107	NDUFS6	NADH dehydrogenase (ubiquinone) Fe-S protein 6	2	1,587	9,8
ENSMUSP00000132927	COX5B	Cytochrome c oxidase, subunit Vb	4	1,509	20,3
ENSMUSP00000015256	SLC25A13	Solute carrier family 25, member 13	7	1,478	3,9
ENSMUSP00000034264	PGLS	6-phosphogluconolactonase	9	1,430	18,2
ENSMUSP00000126699	GLOD4	Glyoxalase domain containing 4	2	1,398	26,8
ENSMUSP00000039472	FGB	Fibrinogen beta chain	6	1,383	18,4
ENSMUSP00000008280	FHL2	Four and a half LIM domains 2	4	1,381	7,9
ENSMUSP00000037348	ACAA2	Acetyl-Coenzyme A acyltransferase 2	9	1,342	13,1
ENSMUSP00000103402	TOMD1	Tropomodulin 1	3	1,337	5,9
ENSMUSP00000025511	RPS14	Ribosomal protein S14	3	0,767	9,5
ENSMUSP00000088349	SELENBP1	Selenium binding protein 1	7	0,753	8,5
ENSMUSP00000039110	SERPB1	Serpine1 mRNA binding protein 1	2	0,751	2,9
ENSMUSP00000038763	PCCA	Propionyl-Coenzyme A carboxylase, alpha polypeptide	5	0,744	4,0
ENSMUSP00000114649	MRPL15	Mitochondrial ribosomal protein L15	5	0,719	9,4
ENSMUSP00000135439	ACAA1A	Acetyl-Coenzyme A acyltransferase 1A	4	0,588	7,8

Table F. Significantly deregulated proteins at 1.0 Gy, 2 years.

ENSEMBL ID	Symbol	Protein	Unique Peptides	Fold changes	Variability [%]
ENSMUSP00000007130	CTNNB1	Catenin (cadherin associated protein), beta 1	4	5,328	23,3
ENSMUSP00000127435	SUCLG2	Succinate-Coenzyme A ligase, GDP-forming	3	4,316	24,9
ENSMUSP00000039172	HSPB6	Heat shock protein, alpha-crystallin-related, B6	7	3,701	19,8
ENSMUSP00000111516	CDH2	Cadherin 2	5	3,301	9,5
ENSMUSP00000022328	LDB3	LIM domain binding 3	6	2,468	12,7
ENSMUSP00000002452	NDUFA11	NADH dehydrogenase (ubiquinone) 1 alpha subcomplex 11	3	2,277	28,6
ENSMUSP00000014080	MYL2	Myosin, light polypeptide 2, regulatory, cardiac	2	2,224	17,9
ENSMUSP00000080994	MT-CO2	Mitochondrially encoded cytochrome c oxidase II	4	2,164	13,3
ENSMUSP00000118601	HK1	Hexokinase 1	5	2,113	9,5
ENSMUSP00000134521	PRDX5	Peroxiredoxin 5	2	2,086	12,8
ENSMUSP00000026743	UQCRC1	Ubiquinol-cytochrome c reductase core protein 1	20	2,074	7,5
ENSMUSP00000106859	TAGLN2	Transgelin 2	3	1,994	24,1
ENSMUSP00000112760	SYNPO2L	Synaptopodin 2-like	11	1,928	16,8
ENSMUSP00000023583	AHSG	Alpha-2-HS-glycoprotein	2	1,915	20,5
ENSMUSP00000114107	NDUFS6	NADH dehydrogenase (ubiquinone) Fe-S protein 6	2	1,834	22,7
ENSMUSP00000039507	MACROD1	MACRO domain containing 1	6	1,746	11,7
ENSMUSP00000029610	HADH	Hydroxyacyl-Coenzyme A dehydrogenase	2	1,721	2,7
ENSMUSP00000042374	HSPB2	Heat shock protein 2	6	1,710	28,8
ENSMUSP00000129776	HRC	Histidine rich calcium binding protein	18	1,706	19,4
ENSMUSP00000122103	SLC25A12	Solute carrier family 25 (Aralar), member 12	16	1,682	26,4
ENSMUSP00000071486	ACTG1	Actin, gamma, cytoplasmic 1	3	1,653	8,2
ENSMUSP00000026289	HSD17B10	Hydroxysteroid (17-beta) dehydrogenase 10	5	1,611	18,3
ENSMUSP00000116919	VDAC1	Voltage-dependent anion channel 1	2	1,606	27,7
ENSMUSP00000048803	PRELP	Proline arginine-rich end leucine-rich repeat	6	1,578	14,0
ENSMUSP00000074600	NDUFS8	NADH dehydrogenase (ubiquinone) Fe-S protein 8	6	1,564	10,8
ENSMUSP00000131316	HSPG2	Perlecan (heparan sulfate proteoglycan 2)	61	1,484	21,8

ENSMUSP00000029266	ANAXA5	Annexin A5	7	1,480	31,0
ENSMUSP00000121557	NDUFV2	NADH dehydrogenase (ubiquinone) flavoprotein 2	7	1,432	9,4
ENSMUSP00000011896	PAGM1	Phosphoglycerate mutase 1	7	1,406	8,1
ENSMUSP00000034264	PGLS	6-phosphogluconolactonase	8	1,385	13,1
ENSMUSP00000038763	PCCA	Propionyl-Coenzyme A carboxylase, alpha polypeptide	8	0,763	16,2
ENSMUSP00000008826	RPL10	Ribosomal protein 10	9	0,754	29,1
ENSMUSP00000101898	UBE2D3	Ubiquitin-conjugating enzyme E2D 3	3	0,754	10,7
ENSMUSP00000106342	GDI2	Guanosine diphosphate (GDP) dissociation inhibitor 2	2	0,749	12,1
ENSMUSP00000039110	SERPB1	Serpine1 mRNA binding protein 1	2	0,746	6,1
ENSMUSP00000088349	SELENBP1	Selenium binding protein 1	8	0,745	14,9
ENSMUSP00000129233	VIM	Vimentin	20	0,645	17,6
ENSMUSP00000028694	PACSIN3	Protein kinase C and casein kinase substrate in neurons 3	7	0,649	24,5
ENSMUSP00000135439	ACAA1A	Acetyl-Coenzyme A acyltransferase 1A	5	0,585	5,2
ENSMUSP00000003066	APOE	Apolipoprotein E	8	0,479	12,4
ENSMUSP00000037206	FKBP1A	FK506 binding protein 1a	3	0,271	27,0
